# EMFE-YOLO: A Lightweight Small Object Detection Model for UAVs

**DOI:** 10.3390/s25165200

**Published:** 2025-08-21

**Authors:** Chengjun Yang, Yan Shen, Lutao Wang

**Affiliations:** School of Computer Science, Chengdu University of Information Technology, Chengdu 610225, China; chengjuny969@gmail.com (C.Y.); wanglt@cuit.edu.cn (L.W.)

**Keywords:** enhanced attention to large-scale features, lightweight small object detection model, multi-scale feature enhancement, unmanned aerial vehicles, YOLOv8

## Abstract

Small object detection in Unmanned Aerial Vehicles’ (UAVs) aerial images faces challenges such as low detection accuracy and complex backgrounds. Meanwhile, it is difficult to deploy the object detection models with large parameters on resource-constrained UAVs. Therefore, a lightweight small object detection model EMFE-YOLO is proposed based on efficient multi-scale feature enhancement by improving YOLOv8s. Firstly, the Enhanced Attention to Large-scale Features (EALF) structure is applied in EMFE-YOLO to focus on large-scale features, improve the detection ability to small objects, and decrease the parameters. Secondly, the efficient multi-scale feature enhancement (EMFE) module is integrated into the backbone of EALF for feature extraction and enhancement. The EMFE module reduces the computational cost, obtains richer contextual information, and mitigates the interference from complex backgrounds. Finally, DySample is employed in the neck of EALF to optimize the upsampling process of features. The EMFE-YOLO is validated on the VisDrone2019-val dataset. Experimental results show that it improves mAP50 and mAP50:95 by 8.5% and 6.3%, respectively, and reduces the parameters by 73% compared to YOLOv8s. These results demonstrate that EMFE-YOLO achieves a good balance between accuracy and efficiency, making it suitable for deployment on UAVs with limited computational resources.

## 1. Introduction

UAVs are increasingly used in various fields such as rescue missions [1], pest detection in agriculture [2], intelligent transportation [3], and animal detection [4], due to their excellent flexibility and mobility. Specifically, object detection is an important part of UAV missions, and it plays a vital role in improving detection efficiency and accuracy. Object detection methods are mainly divided into two categories: traditional methods and deep learning-based methods. Traditional methods identify objects based on handcrafted features. These features rely on human expertise and prior knowledge. It is difficult for them to adapt to complex real-world scenarios. With the advancement of deep learning, object detection methods based on convolutional neural networks (CNNs) have become mainstream. Early CNN-based methods are two-stage approaches represented by the R-CNN [5] series. They generate candidate regions through a Region Proposal Network (RPN) for subsequent classification and regression. Although these methods achieve high detection accuracy in object detection tasks, their heavy computation results in slower inference speeds. Later, one-stage approaches represented by the YOLO [6] series and SSD [7] treat object detection as an end-to-end regression problem. They directly generate predictions from input images without generating candidate boxes, significantly improving inference speed and making them suitable for UAVs with limited computing resources.

In recent years, the YOLO series has been widely applied in the field of object detection. However, the existing YOLO methods still face significant challenges for small object detection in UAV aerial images. On the one hand, these methods are primarily designed for detecting objects of regular sizes, and their ability to extract features from small objects is limited, making it difficult to capture fine-grained information effectively. Meanwhile, small objects in their aerial images are easily disturbed by both complex backgrounds and environmental noise [8,9], resulting in low detection accuracy. On the other hand, the YOLO models still have a large number of parameters, restricting their efficient deployment on UAVs with limited computational resources. Therefore, improving the accuracy of small object detection and achieving model lightweighting and efficient inference remains a critical issue that needs to be addressed.

YOLOv8 [10] is one of the most mature methods in the YOLO series. It incorporates a range of advanced design concepts and has been thoroughly validated through time and practical application, demonstrating strong detection performance and stability. Therefore, this paper improves the YOLOv8s model as the baseline and proposes a lightweight small object detection model for UAV aerial images, called EMFE-YOLO, which is based on efficient multi-scale feature enhancement. To focus on large-scale features and improve the utilization of the detailed information of small objects, the EALF structure is proposed. Specifically, two feature fusion layers with scales of 160×160 and 320×320 are added to the neck to strengthen the representation of large-scale features. A detection head with scale of 160×160 is introduced to improve the detection capability of small objects, meanwhile, the detection head with scale of 20×20 and its redundant network layers are cropped to reduce the parameters. Secondly, the EMFE module is proposed by combining depthwise separable convolution (DSC) [11] with the spatial and channel synergistic attention (SCSA) [12] module. The EMFE module is used in the backbone of EALF structure to lower the computational cost, enhance the expression of contextual information, and mitigate the interference from complex backgrounds. Finally, DySample [13] is employed in the neck of the EALF structure to optimize the upsampling process of feature maps, which enhances the reconstruction of fine details for small objects.

The main contributions of this paper are as follows:

(1) The EALF structure is designed. It improves the detection capability of small objects by focusing on large-scale features while decreasing the parameters.
(2) The EMFE module is proposed to achieve lightweight feature extraction and enhancement, so as to mitigate the interference from complex backgrounds on small objects.
(3) The EMFE-YOLO model is proposed by integrating the EMFE module and the DySample module into the EALF structure. Extensive experiments on the VisDrone2019 dataset [14] demonstrate that EMFE-YOLO achieves outstanding performance.

The rest of this paper is structured as follows: Section 2 reviews the development of the YOLO algorithm and its improvement methods in UAV aerial images. Section 3 provides a detailed introduction to the EMFE-YOLO model, including the working principles of each improvement. Section 4 describes the experimental dataset, environment, evaluation metrics, ablation experiments, comparison experiments, and visualization analysis. Section 5 concludes the paper.

## 2. Related Work

To better understand the current research status of small object detection in aerial images, it is necessary to first review one of the most representative object detection frameworks—the YOLO series. This section provides a systematic overview of the development of YOLO from its initial version to the latest one and summarizes related studies based on YOLO conducted by researchers in this field.

### 2.1. The YOLO Series Methods

The YOLO series are the most commonly used among one-stage methods. YOLO transforms object detection into an end-to-end regression task by dividing the input image into fixed-size grids to predict object categories and bounding box coordinates. YOLO significantly improves inference speed, which makes real-time object detection feasible. YOLO9000 [15] integrates batch normalization and a high-resolution classifier into YOLO, so as to increase detection accuracy. YOLOv3 [16] introduces Darknet-53 as the backbone and employs the Feature Pyramid Network (FPN) for multi-scale feature fusion, which makes it effective at handling complex scenes. YOLOv4 [17] adopts the CSPDarknet53 as the backbone to reduce the computational cost and enhance feature representation. Meanwhile, it introduces Mosaic data enhancement to enrich the spatial and scale variations of the samples and improve the generalization capability of the model. YOLOv5 [18] embeds a lightweight backbone and feature fusion strategy, which improves inference speed. YOLOv6 [19] introduces a self-distillation strategy to enhance model performance, and integrates EfficientRep with Rep-PAN to improve both inference speed and accuracy. YOLOv7 [20] adopts the Efficient Layer Aggregation Network (ELAN) architecture, which optimizes the network structure and computational flow to achieve high detection accuracy with low computational cost. YOLOv8 introduces a new backbone, an anchor-free frame strategy, and an improved loss function to support a wide range of visual tasks. Addressing the information bottleneck problem, YOLOv9 [21] introduces Programmable Gradient Information (PGI) and the Generalized Efficient Layer Aggregation Network (GELAN) to enhance the model’s efficiency and performance. Recently, YOLOv10 [22], YOLOv11 [23], and YOLOv12 [24] have been released, which optimize the network architecture and inference strategies to provide better support for real-time object detection.

### 2.2. The Improved YOLO Methodology

Due to the outstanding performance of the YOLO series in real-time object detection tasks, researchers have explored their potential in UAV aerial images by introducing various enhancement mechanisms.

To solve the problems of large changes in object scale and the motion blur on the densely packed objects in UAV aerial images, Zhu et al. [25] proposed TPH-YOLOv5, which introduced a transformer predictor head to capture long-distance feature dependencies and enhance the detection capability of small objects. Although TPH-YOLOv5 exhibits higher accuracy in small object detection, the transformer predictor head significantly increases the computational cost. To address this issue and enhance the generalization capability of TPH-YOLOv5, Zhao et al. [26] proposed TPH-YOLOv5++, which integrates the Cross-Layer Asymmetric Transformer (CA-Trans) and Sparse Local Attention (SLA) mechanisms to enrich small object features while reducing computational overhead. Zhao et al. [27] proposed MS-YOLOv7, which addresses high-density object detection by integrating Swin Transformer units with the SPPFS module. Zeng et al. [28] proposed a binary K-means anchor generation algorithm to address the imbalance in object sizes between datasets and real-world scenarios. Li et al. [29] integrated GhostBlockV2 and Bi-PAN-FPN into YOLOv8 to reduce parameters and enhance feature fusion, while introducing WiseIoU to optimize the performance of bounding box regression. Lou et al. [30] proposed DC-YOLOv8, which integrates the MDC downsampling module and the DC module to capture richer contextual information, so as to effectively improve the detection accuracy of small objects. Li et al. [31] proposed SOD-YOLO, which optimized the feature extraction efficiency by designing the the Receptive Field Convolutional Block Attention Module (RFCBAM) to replace the downsampling of the backbone network. Meanwhile, the Balanced Spatial and Semantic Information Fusion Pyramid Network (BSSI-FPN) structure was proposed to improve the detection capability of small objects by combining large-scale features with deep semantic features. Hu et al. [32] proposed LW-YOLOv8, which improved the detection accuracy of small objects and reduced the parameters by introducing a lightweight VanillaNet architecture and an Asymptotic Feature Pyramid Network (AFPN). Zhao et al. [33] proposed the YOLO-DroneMS model to address the challenges of large differences in object scales and complex backgrounds. They incorporated Large Separable Kernel Attention (LSKA) in the SPPF module to strengthen the representation of multi-scale features. They introduced the C2f-iRMB-DRB structure to enhance the feature extraction capability of the backbone. Additionally, the DySample module and WIoUv3 loss function were integrated to boost the detection performance of small objects further. Li et al. [34] designed a Multi-head Channel and Spatial Trans-Attention (MCSTA) module, which achieves remote pixel interaction from both channel and spatial dimensions to complete the attention feature capture function.

## 3. Methods

To address the many challenges of small object detection in aerial images, this paper proposes the EMFE-YOLO method. This section provides a detailed introduction to its core structure and key improvements from four perspectives: Overview, EALF Structure, EMFE Module, and DySample.

### 3.1. Overview

**Figure 1:**
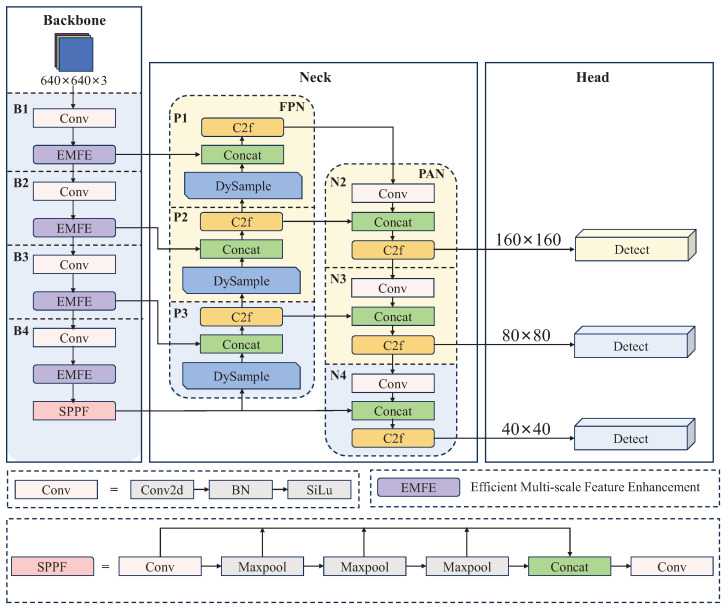
Proposed EMFE-YOLO model.

The EMFE-YOLO model is shown in Figure 1. Notably, the overall structure of EMFE-YOLO adopts the EALF structure, which strengthens attention to large-scale features, enhances the detection ability to small objects, and reduces the parameters. Secondly, the EMFE module is used in the backbone to achieve multi-scale feature extraction and enhancement on the feature maps generated after four downsampling stages. This approach efficiently captures richer contextual detail information, reduces the impact of complex background on small objects, and decreases the computational cost. Finally, the DySample module is applied in the neck to improve the flexibility of feature reconstruction. By integrating the EALF structure, EMFE module, and DySample module, EMFE-YOLO enhances the detection capability of small objects while maintaining low computational complexity and parameters. This provides an efficient, lightweight, and accurate solution for small object detection in UAVs’ aerial images.

### 3.2. EALF Structure

**Figure 2:**
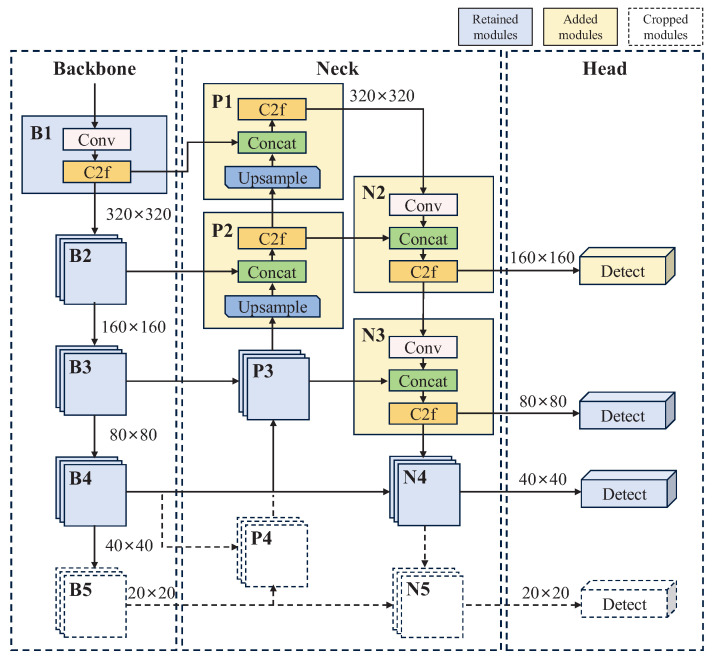
Proposed EALF structure. The blue boxes indicate the parts that were kept in YOLOv8, the yellow boxes indicate the parts that were added, and the white boxes indicate the parts that were cropped out.

The YOLOv8 backbone employs five downsampling operations to extract features. The feature maps from layers B3, B4, and B5 are fed into the neck for multi-scale feature fusion. With an input image size of 640×640, the sizes of the feature fusion layers at the neck and the final detection feature maps are 80×80, 40×40, and 20×20, respectively. However, most targets in UAV aerial images are small objects. Large-scale features contain rich spatial details and are essential for detecting them. Consequently, enhancing the utilization of large-scale features can improve the ability to locate and recognize small objects. Therefore, we optimize the network structure of YOLOv8 and propose the EALF structure to better adapt to the small object detection task in UAV aerial images. The specific design of the EALF structure is shown in Figure 2.

In the neck, P1, P2, N2, and N3 feature fusion layers are added to efficiently extract and fuse feature maps at 320×320 and 160×160 scales, which enhances the representation of large-scale features. The P1 layer receives feature information from the B1 layer of the backbone and is concatenated with the upsampling output from the P2 layer (Equation (1)). This design ensures that the P1 layer retains the highest resolution, enhancing its ability to capture spatial detail information. The P2 layer accepts feature information from the B2 layer and is concatenated with the upsampling output from the P3 layer (Equation (2)). In contrast to the P1 layer, the P2 layer introduces some semantic features, which strengthens the ability to differentiate between small object categories and compensates for the lack of semantic information in large-scale features. Layer N2 accepts feature information from Layer P2 and splices it with the downsampled output of Layer P1 (Equation (3)); Layer N3 accepts feature information from Layer P3 and splices it with the downsampled output of Layer N2 (Equation (4)). By utilizing large-scale features fully, the sensitivity of the model to spatial detail information is enhanced, so as to mitigate the loss of fine-grained information in deeper layers.

In the detection head, a detection head with scale of 160×160 is introduced and connected to the N2 layer, which improves the ability to locate and recognize small objects. At the same time, the detection head with scale of 20×20 and its redundant layers (B5, P4, N5) are cropped to decrease the parameters. The neck structure is simplified to achieve lightweight feature fusion without reducing detection accuracy.

(1) $$\begin{array}{cc} P1 & =Concat(B1,UpSample(P2)) \end{array}$$

(2) $$\begin{array}{cc} P2 & =Concat(B2,UpSample(P3)) \end{array}$$

(3) $$\begin{array}{cc} N2 & =Concat(P2,Conv_{\left(3\times 3\right)}\left(P1\right)) \end{array}$$

(4) $$\begin{array}{cc} N3 & =Concat(P3,Conv_{\left(3\times 3\right)}\left(N2\right)) \end{array}$$

### 3.3. EMFE Module

**Figure 3:**
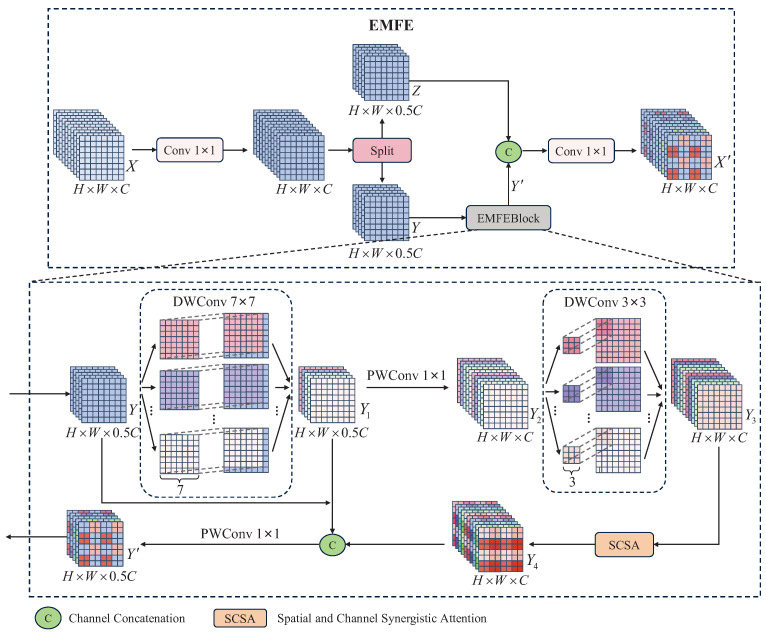
Proposed EMFE module.

Small objects in UAV aerial images usually have a small pixel area, limited feature representation, and are easily affected by complex backgrounds. Although the EALF structure can improve the detection accuracy of small objects, its ability to distinguish features is still limited when facing the interference from background noise. To address this issue, this paper proposes the EMFE module, which is centered around the EMFEBlock and combines convolutional layers with residual connections. The EMFE structure is shown in Figure 3.

Start with an input feature map $X\in R^{H\times W\times C}$ (where *H* and *W* are the height and width of the feature map, and *C* is the number of channels). Firstly, a 1×1 convolution is used to transform the features of *X*. Then, the feature map is split along the channel dimension into two equal parts, $Y\in R^{H\times W\times 0.5C}$ and $Z\in R^{H\times W\times 0.5C}$. *Y* is fed into the EMFEBlock module for feature extraction and enhancement to obtain $Y^{\prime }\in R^{H\times W\times 0.5C}$, and the other part of *Z* is fed into the Concat module for splicing with $Y^{\prime }$. Finally, the spliced result is used as the input to obtain the output result $X^{\prime }\in R^{H\times W\times C}$ by a 1×1 convolution again. The computational process can be formulated as follows:

(5) $$\begin{array}{cc} Y,Z & =Split(Conv_{\left(1\times 1\right)}\left(X\right)) \end{array}$$

(6) $$\begin{array}{cc} Y^{\prime } & =EMFEBlock(Y) \end{array}$$

(7) $$\begin{array}{cc} X^{\prime } & =Conv_{\left(1\times 1\right)}\left(Concat\left(Y^{\prime },Z\right)\right) \end{array}$$

Figure 3 shows the working process of EMFEBlock. Begin with the input $Y\in R^{H\times W\times 0.5C}$. Firstly, a 7×7 depthwise convolution (DWConv) is used to capture a wide range of contextual information in the low-dimensional feature space to obtain $Y_{1}\in R^{H\times W\times 0.5C}$. Secondly, a 1×1 pointwise convolution (PWConv) is applied to extend the number of feature channels to twice the input to obtain $Y_{2}\in R^{H\times W\times C}$, which enhances the richness of feature expression.

Then, a 3×3 DWConv is again used to further extract deep features in the high-dimensional feature space to obtain the output $Y_{3}\in R^{H\times W\times C}$, which enhances the expression of fine-grained information. Subsequently, $Y_{3}$ is fed to the SCSA module for a feature enhancement to obtain $Y_{4}\in R^{H\times W\times C}$, which suppresses background noise through the synergy of spatial and channel attention. Finally, $Y_{4}$ is spliced with the low-dimensional feature maps *Y* and $Y_{1}$ along the channel, and $Y^{\prime }\in R^{H\times W\times 0.5C}$ is obtained by integrating and downscaling the features at different scales through a 1×1 PWConv, which achieves unified representation and effective fusion of multi-scale information. The process can be formulated as follows:

(8) $$\begin{array}{cc} Y_{1} & =DWConv_{\left(7\times 7\right)}\left(Y\right) \end{array}$$

(9) $$\begin{array}{cc} Y_{2} & =PWConv_{\left(1\times 1\right)}\left(Y_{1}\right) \end{array}$$

(10) $$\begin{array}{cc} Y_{3} & =DWConv_{\left(3\times 3\right)}\left(Y_{2}\right) \end{array}$$

(11) $$\begin{array}{cc} Y_{4} & =SCSA(Y_{3}) \end{array}$$

(12) $$\begin{array}{cc} Y^{\prime } & =PWConv_{\left(1\times 1\right)}\left(Concat\left(Y,Y_{1},Y_{4}\right)\right) \end{array}$$

**Figure 4:**
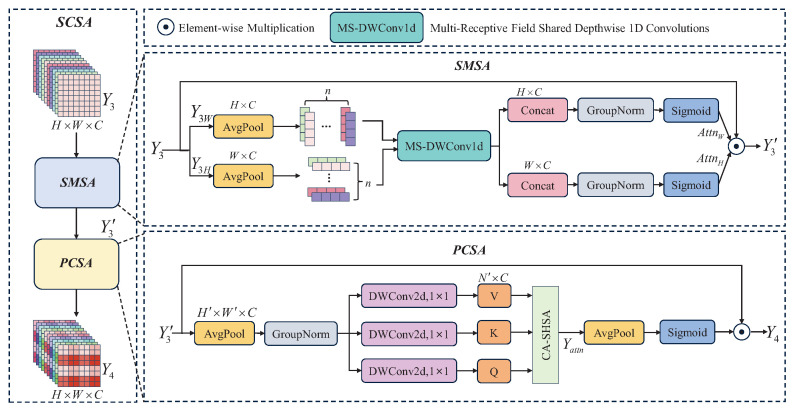
SCSA module structure diagram. Spatial attention and channel attention are extracted from the two sub-modules SMSA and PCSA, respectively.

SCSA enhances feature representation at both spatial and channel levels to provide more comprehensive and detailed support for subsequent detection. SCSA consists of shared Multi-Semantic Spatial Attention (SMSA) and Progressive Channel-wise Self-Attention (PCSA). SMSA integrates multi-semantic information to generate comprehensive spatial feature representations, which provide valuable spatial prior information to PCSA and guide it to adjust channel feature weights more accurately. PCSA implements feature interaction at the channel level through a single-head self-attention mechanism, alleviating conflicts between multi-semantic features. The SCSA computation process is shown in Figure 4. SMSA decomposes the input feature map $Y_{3}\in R^{H\times W\times C}$ into two unidirectional 1D sequence structures $Y_{3H}\in R^{W\times C}$ and $Y_{3W}\in R^{H\times C}$ along the height (*H*) and width (*W*) dimensions, and uniformly divides them into n equally sized sub-features. Subsequently, the MS-DWConv1d module is used to extract the semantic information of the sub-features. Finally, $Y_{3}^{\prime }$ is generated by concatenating, applying Group Normalization (GN), and using the Sigmoid activation function on the output of MS-DWConv1d. The process can be formulated as follows:

(13) $$Y_{3}^{\prime }=\mathrm{SMSA}\left(Y_{3}\right)=Attn_{H}\times Attn_{W}\times Y_{3}$$

where $Y_{3}^{\prime }\in R^{H\times W\times C}$. $Attn_{H}$ and $Attn_{W}$ denote the attention in the height and width dimensions, respectively. Then, $Y_{3}^{\prime }$ is taken as input into the PCSA for average pooling to compress the dimensions as $H^{\prime }\times W^{\prime }\times C$. Subsequently, three vectors of query ($Q\in R^{N^{\prime }\times C}$), key ($K\in R^{N^{\prime }\times C}$), and value ($V\in R^{N^{\prime }\times C}$) are generated by using DWConv, and the channel attention $Y_{\mathrm{attn}}$ is obtained by the Channel-wise Single-Head Self-Attention (CA-SHSA). $Y_{\mathrm{attn}}$ can be calculated by the following equation:

(14) $$Y_{\mathrm{attn}}=Attn\left(Q,K,V\right)=Soft\max\left(\frac{QK^{T}}{\sqrt{C}}\right)V$$

$Y_{4}$ is obtained by average pooling operation and Sigmoid activation function:

(15) $$Y_{4}=PCSA\left(Y_{3}^{\prime }\right)=Y_{3}^{\prime }\times \sigma \left(AvgPool\left(Y_{attn}\right)\right)$$

EMFE achieves efficient feature extraction and fusion through depthwise separable convolutions (DSCs) at different scales. EMFE also incorporates the SCSA module to enhance the expression of contextual information. In addition, it introduces a residual connection to optimize gradient to enhance the stability of feature representation and the utilization of multi-scale information.

### 3.4. DySample

Upsampling restores spatial information by reconstructing the features. YOLOv8 constructs a bidirectional cross-scale feature fusion mechanism through a cascade architecture of Feature Pyramid Network (FPN) and Path Aggregation Network (PAN), where the upsampling module is responsible for gradually aligning spatial features with semantic features. The default upsampling method in YOLOv8 is nearest neighbor interpolation. Although this method is simple, it lacks adaptability to input content and may lose details when dealing with small objects or complex backgrounds.

**Figure 5:**
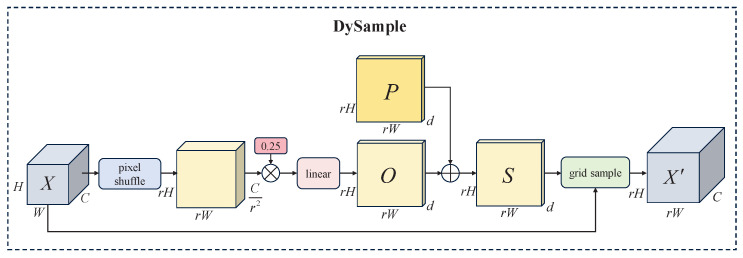
DySample module structure.

Therefore, the DySample module is used for dynamic upsampling in the neck. DySample makes upsampling more flexible and accurate by formulating upsampling from the perspective of point sampling. Compared to kernel-based upsamplers (e.g., CARAFE [35] and FADE [36]), DySample avoids a higher computational cost. Its sampling process is shown in Figure 5. Given an input feature map $X\in R^{H\times W\times C}$ (where *C* denotes the dimension of *X*, and *H* and *W* denote the height and width of *X*, respectively), an upsampling scale factor *r* and a static range factor of 0.25, pixel shuffle is applied to obtain the output of size $rH\times rW\times \frac{C}{r^{2}}$. (We conducted preliminary experiments by setting the static range factor *r* to 0.1, 0.25, 0.5, and 1 for comparison. The results showed that the model achieves the best performance when *r* = 0.25, which is therefore adopted in this study.) The output is multiplied by the static range factor 0.25 and passed through a linear layer to obtain the offset $O\in R^{rH\times rW\times d}$ (where *d* = 2, which represents the *x* and *y* coordinates of the sampling point). Finally, *O* is added to the original sampling grid *P* to obtain the sampling set $S\in R^{rH\times rW\times d}$. This process can be formulated as follows:

(16) $$\begin{array}{cc} O & =0.25linear(X) \end{array}$$

(17) $$\begin{array}{cc} S & =O+P \end{array}$$

Finally, *X* and *S* are fed into the grid_sample function, and *X* is resampled by using bilinear interpolation to obtain $X^{\prime }\in R^{rH\times rW\times C}$. This process can be expressed as follows:

(18) $$X^{\prime }=grid\_sample\left(X,S\right)$$

DySample can adaptively select key information for sampling, which generates higher-resolution features with greater expressive power. This helps mitigate the detection difficulties caused by low resolution and limited feature information in small objects.

## 4. Experiments

This section designs a series of experiments to comprehensively evaluate the detection performance of the EMFE-YOLO method. The experiments cover the basic setup (dataset, environment, and parameter configuration), evaluation indicators, and multiple empirical analyses (ablation experiments, comparison experiments, and visualizations). The following subsections provide a detailed description of each part.

### 4.1. Dataset

**Figure 6:**
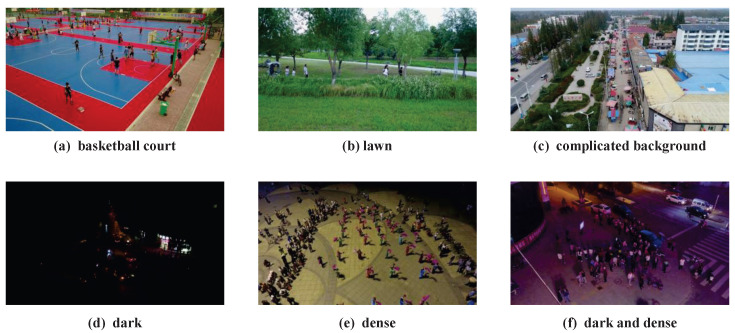
Some examples from the VisDrone2019 dataset.

The VisDrone2019 dataset was collected and annotated by the AISKYEYE team from Tianjin University. This dataset consists of 288 video clips, 261,908 video frames, and 10,209 still images. There are 6471 images in the training set, 548 images in the validation set, and 1610 images in the test set. These data were collected by UAV cameras and cover a wide range of areas. They include footage from both urban and rural environments across 14 cities in China, featuring 10 different object categories such as pedestrians, people, and cars. The scene density ranges from sparse to crowded, which fully demonstrates the wide adaptability of UAV applications and reflects the challenges of object detection in UAV aerial images, meeting the experimental requirements of this study. Some data samples are shown in Figure 6.

### 4.2. Experimental Environment and Parameter Configuration

As shown in Table 1, the experiments in this paper are based on the Ubuntu 18.04 operating system, using Python 3.8, PyTorch 1.12.1+cu113, and Cuda 11.3 as the deep learning experiment environment. An NVIDIA GeForce RTX 3090 graphics card is used as hardware for the experiments. The relevant hyperparameters are configured as follows: the number of training rounds is 300, the batch size is 2, the input image size is uniformly scaled to 640×640, and data enhancement is turned on. The hyperparameters used for all experiments in this paper remain the same for training and validation.

### 4.3. Evaluation Indicators

To validate the performance of EMFE-YOLO, the experiments are evaluated in terms of both detection accuracy and model lightweighting. The detection indicators include precision (P), recall (R), mean average precision (mAP), F1 score, parameters (M), GFLOPs, and FPS. precision represents the proportion of actual positive samples among those predicted as positive by the model (Equation (19)). Recall indicates the proportion of successfully detected objects among all actual positive samples (Equation (20)).

(19) $$\begin{array}{cc} P & =\frac{TP}{TP+FP} \end{array}$$

(20) $$\begin{array}{cc} R & =\frac{TP}{TP+FN} \end{array}$$

where TP refers to the number of true positive samples, FP refers to the number of false positive samples, and FN refers to the number of false negative samples. Higher precision indicates more accurate predictions for positive samples, which reduces the number of false positives. Higher recall reflects more comprehensive detection of positive samples, resulting in fewer false negatives.

Average precision (AP) is an important metric for evaluating the performance of single-class object detection (Equation (21)). The mAP is the average of AP over all categories and is used to indicate overall detection performance (Equation (22)). The F1 score is the harmonic mean of precision and recall (Equation (23)). The parameters, GFLOPs, and FPS are important metrics for evaluating the lightweight design of object detection models. Specifically, the number of parameters reflects the model’s storage complexity, GFLOPs indicate its computational complexity, and FPS represents the model’s inference speed in practical use.

(21) $$\begin{array}{cc} AP & =\int_{0}^{1}P(R)dR \end{array}$$

(22) $$\begin{array}{cc} mAP & =\frac{1}{C}\sum_{C=1}^{C}AP_{C} \end{array}$$

(23) $$\begin{array}{cc} F1 & =\frac{2\times P\times R}{P+R} \end{array}$$

where *P* and *R* in Equations (21) and (23) are consistent with those in Equations (19) and (20). In Equation (22), *C* denotes the number of categories, and $AP_{C}$ represents the average precision of the c-th category.

### 4.4. Ablation Experiments

To validate the effectiveness of EMFE-YOLO, this section designs a series of ablation experiments on the VisDrone2019-val dataset, as shown in Table 2. The effects of each improvement are evaluated and analyzed based on various evaluation metrics, including precision, recall, mAP50, mAP50:95, parameters, GFLOPs, and FPS.

Under the same experimental conditions, the mAP50 of YOLOv8s is 38.4%. After adopting the EALF structure, its mAP50 and mAP50:95 increase to 44% and 27.5%, respectively, which represent significant improvements of 5.6% and 4.7% compared to YOLOv8s. Meanwhile, the model parameters are reduced to 3.4 M, while the GFLOPs increase to 35.7. This result indicates that focusing on large-scale features can effectively enhance the detection capability of small objects, while pruning deep redundant layers can significantly reduce the parameters. However, due to the high resolution of large-scale feature maps, additional computational overhead is introduced during processing, resulting in a slight increase in overall computational complexity. Consequently, the FPS drops from 172 to 143.

After incorporating both the EALF structure and the EMFE module, the mAP50 increases to 45.9%, the model parameters are reduced to 3M, and GFLOPs drop to 33.1. This indicates that EMFE enhances detection accuracy while optimizing computational efficiency. Furthermore, when EMFE is integrated into YOLOv8 alone, the performance improvement is minimal, with mAP50 only increasing by 0.1%. This suggests that EMFE needs to work in conjunction with the EALF architecture to fully realize its potential advantages.

The combination of DySample with EALF and EMFE has had a positive impact on the model’s performance. As shown in the table, after integrating DySample, the mAP50 increases to 46.9%, and the mAP50:95 rises to 29.1%. Compared to using only EALF and EMFE, this further improves the detection capability of small objects. Moreover, DySample optimizes computational efficiency while maintaining high accuracy, keeping the GFLOPs at 33.1 and the parameters at 3M.

In summary, EMFE-YOLO achieves the best performance with an mAP50 of 46.9% and an mAP50:95 of 29.1%. Despite the reduction in FPS from 172 to 121, the model size is compressed to 3 M, achieving a balance between accuracy and efficiency.

### 4.5. Comparison Experiment

In order to further evaluate the performance of EMFE-YOLO, four sets of comparison experiments are designed in this section to comprehensively analyze and validate its performance. The first set of experiments aims to analyze the detection performance of the EALF structure across objects of different scales. The second set evaluates the robustness of EMFE-YOLO under environmental noise interference. The third set compares the detection performance between EMFE-YOLO and YOLOv8s. The fourth set involves a comparison between EMFE-YOLO and other object detection models.

#### 4.5.1. Comparative Analysis of the EALF Structure on Different Object Scales

The EALF structure enhances the detection capability of small objects by focusing on large-scale features while also reducing the parameters. To further verify its effectiveness across objects of different scales, this section follows the MS COCO [37] dataset’s classification criteria and divides all objects in the VisDrone2019-val dataset into three categories: small (area < 32 × 32), medium (32 × 32 ≤ area ≤ 96 × 96), and large (area > 96 × 96). YOLOv8s and the EALF structure are used for inference on the validation set, and their detection performance at different object scales is shown in Table 3.

According to the experimental results, the VisDrone2019-val dataset contains 38,759 objects in total, among which the proportion of small, medium, and large objects is approximately 69%, 29%, and 2%, respectively. In terms of detection performance at different object scales, the EALF detects 3301 more small objects, 122 more medium objects, and 24 more large objects than the baseline YOLOv8s, demonstrating its superior ability to adapt to varying object scales. It is worth noting that although the EALF structure removes the detection head with scale of 20×20 and its redundant network layers, it does not weaken the model’s ability to detect large objects and even shows a slight improvement. This further confirms the effectiveness of the EALF structure.

#### 4.5.2. Comparative Analysis of Robustness Under Environmental Noise Interference

Noise interference is one of the key challenges in object detection, and it is particularly prominent in UAV aerial images [38]. The proposed EMFE module enhances the ability to capture contextual information for small objects, mitigating the impact of complex background noise effectively. To further evaluate the robustness of EMFE-YOLO under environmental noise, this section introduces Gaussian white noise (with SNRs of 0 dB, 10 dB, 15 dB, and 20 dB) into the VisDrone2019-val dataset. The experimental results are shown in Table 4. The experimental results show that EMFE-YOLO consistently outperforms the baseline YOLOv8s in detection performance under varying levels of Gaussian white noise. Although both models exhibit a downward trend in mAP50 as noise intensity increases, EMFE-YOLO shows a smaller performance drop, indicating stronger robustness.

#### 4.5.3. Comparison with the Baseline YOLOv8s Model

This section presents a comparative analysis between the baseline YOLOv8s model and EMFE-YOLO on the VisDrone2019-val dataset (Table 5) and the VisDrone2019-test dataset (Table 6). As shown in Table 5, EMFE-YOLO improves the mAP50 for every category on the VisDrone2019-val dataset, resulting in an overall mAP50 increase of 8.5%. Notably, the mAP50 for the pedestrian and person improves significantly, which indicates that EMFE-YOLO has a stronger ability to detect small and densely objects. As shown in Table 6, the overall mAP50 value of EMFE-YOLO on the VisDrone2019-test dataset increased by 6.2%. This further validates the powerful performance of EMFE-YOLO.

**Figure 7:**
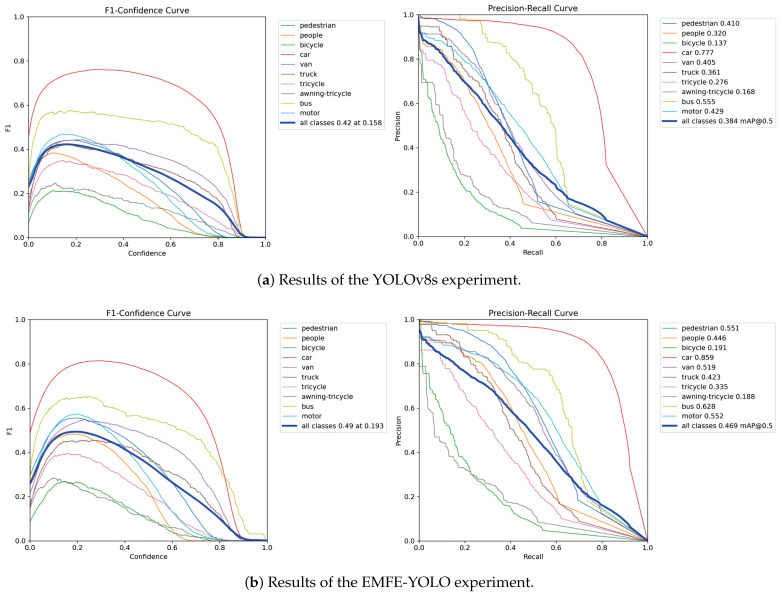
Experimental results of YOLOv8s and EMFE-YOLO on VisDrone2019-val.

Figure 7 shows the experimental results of YOLOv8s and EMFE-YOLO on the VisDrone2019-val dataset. As can be seen from the precision–recall curve in the figure, EMFE-YOLO covers a larger range, which indicates that the model has a better balance between precision and recall. In the F1–confidence curve, EMFE-YOLO shows a higher F1 score, verifying the effectiveness of the model.

#### 4.5.4. Comparison with Other Object Detection Models

This section selects other object detection models (Faster RCNN [39], CenterNet [40], YOLOv8n, YOLOv8l, YOLOv9s, YOLOv10s, YOLOv11s, YOLOv12s, TPH-YOLOv5, RT-DETR-R18 [41], YOLO-ERF-L [42], UAV-YOLOv8, Drone-YOLO [43], PVswin-YOLOv8s [44], and TA-YOLO-s) for comparative experiments with EMFE-YOLO. The analysis and evaluation are based on mAP50, mAP50:95, and model parameters. The experimental results are shown in Table 7.

From the experimental results, it can be observed that EMFE-YOLO demonstrates a comprehensive performance advantage on the VisDrone2019-val dataset. EMFE-YOLO achieves an mAP50 of 46.9%, which is 3.9% higher than YOLOv8l, 6.9% higher than YOLOv9s, and only 0.1% lower than UAV-YOLOv8. Under the more stringent mAP50:95 metric, EMFE-YOLO reaches 29.1%, surpassing YOLOv8l by 2.6%, TA-YOLO-s by 1.4%, and Faster R-CNN by 7.2%, while maintaining comparable performance to UAV-YOLOv8. Compared to TPH-YOLOv5, RT-DETR-R18, and YOLO-ERF-L, EMFE-YOLO also demonstrates superior detection performance, improving mAP50 by 5.2%, 4.4%, and 3.2%, respectively, while significantly reducing the number of parameters. Regarding model parameters, EMFE-YOLO contains only 3 million parameters, which is equivalent to 13.9% of UAV-YOLOv8’s parameters, and much lower than CenterNet’s 5% and RT-DETR-R18’s 15.7%. Compared with other lightweight methods such as Drone-YOLO and PVswin-YOLOv8s, EMFE-YOLO achieves a higher mAP with fewer parameters. These results indicate that EMFE-YOLO achieves a better balance between accuracy and efficiency, making it suitable for deployment on resource-constrained UAVs.

### 4.6. Visualization

To demonstrate the effect of the EMFE-YOLO model more intuitively, this section comprehensively evaluates its detection capability in two ways. The first involves a quantitative analysis of the confusion matrix to reveal the model’s accuracy in detecting different object categories. The second approach combines inference results from real UAV aerial images to visually validate the model’s detection performance in complex scenarios.

**Figure 8:**
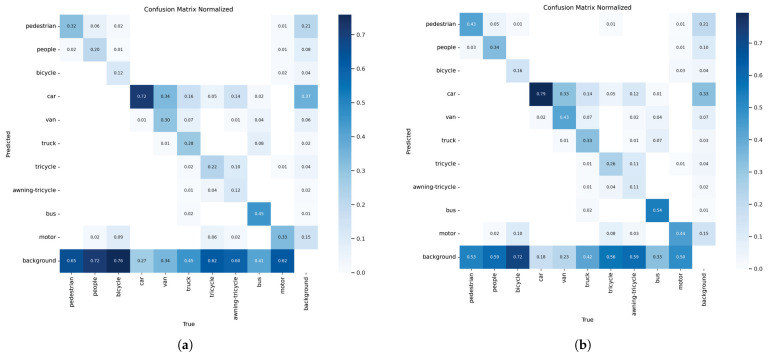
(**a**) YOLOv8s confusion matrix (**b**) EMFE-YOLO confusion matrix.

As shown in Figure 8, the confusion matrices of YOLOv8s and EMFE-YOLO are constructed to demonstrate the model’s classification performance on ten different categories in the dataset. The rows and columns of the confusion matrix represent the true and predicted categories, respectively. The values on the main diagonal show the percentage of correct classifications for each category. Darker colors indicate higher accuracy. The other areas represent incorrect predictions, with deeper colors showing a higher percentage of errors for the corresponding category. In Figure 8, the color of the main diagonal elements in the EMFE-YOLO confusion matrix is noticeably darker than YOLOv8s. Additionally, each main diagonal element’s value is higher than the corresponding value in YOLOv8s, indicating that the EMFE-YOLO model has improved accuracy in predicting all ten categories. Among them, the three categories with the greatest improvement were pedestrian, people, and motor, which were improved by 11%, 14%, and 11%, respectively. The last row of the EMFE-YOLO confusion matrix shows a lighter color, indicating that the model’s probability of misclassifying objects as background has been significantly reduced. This further validates its robustness in complex scenarios.

**Figure 9:**
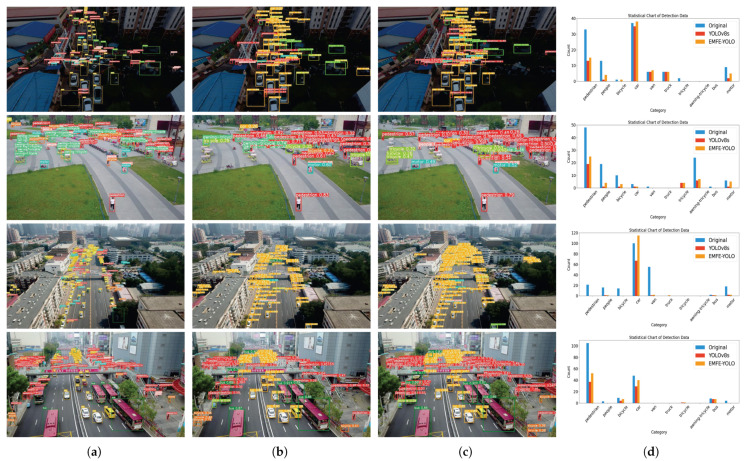
Detection results of YOLOv8s and EMFE-YOLO on real UAV aerial images: (**a**) ground truth annotations, (**b**) detection results produced by YOLOv8s, (**c**) detection results produced by EMFE-YOLO, (**d**) statistical table of the detection results.

Figure 9 illustrates the detection results of YOLOv8s and EMFE-YOLO on real UAV aerial images. Specifically, Figure 9a presents the ground truth annotations, Figure 9b shows the detection results produced by YOLOv8s, and Figure 9c shows the results obtained by EMFE-YOLO. Notably, Figure 9d provides a statistical comparison of detection counts across different categories for the ground truth, YOLOv8s, and EMFE-YOLO. The x-axis lists ten categories from the VisDrone2019 dataset, while the y-axis shows the number of detections from the ground truth and the models. Compared to YOLOv8s, EMFE-YOLO demonstrates a significant performance advantage. It is capable of detecting a greater number of objects, particularly excelling in the detection of pedestrians and cars. This indicates that EMFE-YOLO possesses a stronger ability to handle dense objects and small objects.

**Figure 10:**
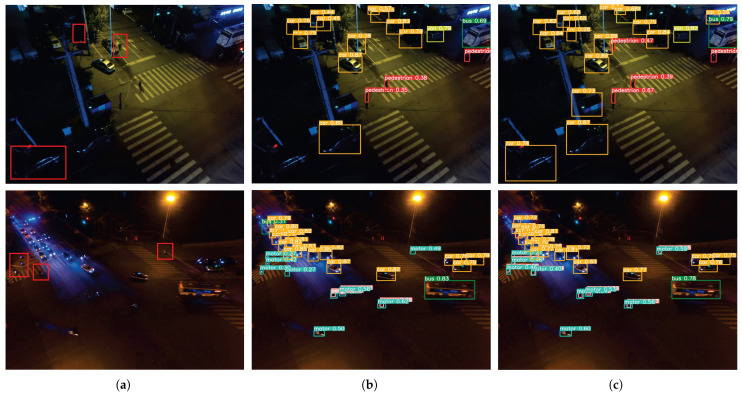
Experimental results of EMFE-YOLO and YOLOv8s on aerial images containing difficult samples. (**a**) Original images, with red boxes highlighting difficult cases such as occluded or motion-blurred objects. (**b**) Detection results of YOLOv8s. (**c**) Detection results of EMFE-YOLO.

Figure 10 compares the detection performance of EMFE-YOLO and YOLOv8s on aerial images containing difficult samples. Figure 10a shows the original images, where red boxes highlight typical difficult cases, such as occluded cars and motion-blurred objects. Figure 10b presents the results of YOLOv8s, while Figure 10c shows the results of EMFE-YOLO. The results demonstrate that YOLOv8s tends to miss detections or assign low confidence scores when dealing with partially occluded or blurred objects. For instance, most targets marked by red boxes are either not detected or assigned low confidence by YOLOv8s. In contrast, EMFE-YOLO shows stronger robustness under the same conditions, successfully detecting small targets that are occluded or blurry, and assigning them higher confidence scores. This further confirms the advantages of EMFE-YOLO, especially its adaptability to challenging conditions such as low resolution, poor lighting, and occlusion.

## 5. Conclusions and Discussion

To address the challenges of small object size and weak feature information in UAV aerial images, the EALF structure is designed to focus on large-scale features, significantly improving small object detection performance. At the same time, the large object detection layer with scale of 20 × 20 is cropped to reduce the model’s parameters effectively. Next, the EMFE module is integrated into the backbone of EALF. EMFE enhances the model’s ability to capture contextual information for small objects and effectively suppresses interference from complex backgrounds. Meanwhile, the EMFE module achieves a lightweight design without compromising detection accuracy by combining the advantages of depthwise separable convolution. Finally, the DySample module is introduced into the neck to optimize the upsampling process. Extensive experiments on the VisDrone2019 dataset demonstrate that EMFE-YOLO achieves a good balance between accuracy and efficiency, reaching 46.9% mAP50 with only 3 million parameters. Compared to existing lightweight detectors such as Drone-YOLO, PVswin-YOLOv8s, and TA-YOLO-s, EMFE-YOLO improves mAP50 by 4.1%, 3.6%, and 1.5%, respectively. This clearly shows its performance advantages in small object detection.

Despite the significant results, there are still limitations worth discussing. On the one hand, the EALF structure significantly improves small object detection accuracy. However, processing large-scale features increases the computational cost, which slows down the model’s inference speed. On the other hand, although EMFE-YOLO demonstrates stronger robustness than YOLOv8s in environmental noise experiments, it still shows a performance drop. Therefore, future research will focus on two directions. First, we will combine techniques such as model distillation and structural pruning to further reduce the model size and computational complexity, thereby improving inference efficiency. Second, we will introduce strategies like noise-enhanced training, multi-task learning, or adversarial training to enhance the model’s adaptability to complex environmental noise, improving its stability and robustness.

## Figures and Tables

**Table 1:** Experimental environment and parameter configuration.

Name	Configuration
Operating System	Ubuntu 18.04
GPU	NVIDIA GeForce RTX 3090
Programming language	Python 3.8
Deep-learning framework	PyTorch 1.12.1+cu113
Epochs	300
Batch Size	2
Image Size	640×640

**Table 2:** Ablation experiment results on VisDrone2019-val.

Baseline	EALF	EMFE	DySample	P	R	mAP50 (%)	mAP50:95 (%)	Param (M)	GFLOPs	FPS
✓				49.6	37.5	38.4	22.8	11.1	28.7	172
✓	✓			53.3	42.1	44.0	27.5	3.4	35.7	143
✓		✓		49.4	37.7	38.5	22.8	10.7	26.0	151
✓			✓	52.2	38.3	39.7	23.8	11.1	28.5	156
✓	✓	✓		54.7	43.9	45.9	28.2	3.0	33.1	128
✓	✓		✓	55.3	43.7	45.5	28.4	3.4	35.2	135
✓	✓	✓	✓	55.2	45.3	46.9	29.1	3.0	33.1	121

**Table 3:** Experimental results of the EALF structure on different object scales.

Scale	Definition (pixels)	Ground Truth	YOLOv8s	EALF
Small	area < 32 × 32	26,586	11,929	15,230
Medium	32 × 32 ≤ area ≤ 96 × 96	11,105	10,531	10,653
Large	area > 96 × 96	1068	1043	1067
Total	-	38,759	23,503	26,950

**Table 4:** Experimental results under environmental noise interference.

Noise Level (dB)	YOLOv8s (mAP50 (%))	EMFE-YOLO (mAP50 (%))
0	38.4 (↓0%)	46.9 (↓0%)
10	35.1 (↓8.6%)	43.2 (↓7.9%)
15	31.9 (↓9.1%)	39.4 (↓8.9%)
20	28.3 (↓11.3%)	35.2 (↓10.7%)

**Table 5:** Experimental results of YOLOv8s and EMFE-YOLO on VisDrone2019-val.

Methods	Pedestrian	People	Bicycle	Car	Van	Truck	Tricycle	Awning-Tricycle	Bus	Motor	mAP50 (%)
YOLOv8s	41	32	13.7	77.7	40.5	36.1	27.6	16.8	55.5	42.9	38.4
EMFE-YOLO (Ours)	55.1	44.6	19.1	85.9	51.9	42.3	33.5	18.8	62.8	55.2	46.9

**Table 6:** Experimental results of YOLOv8s and EMFE-YOLO on VisDrone2019-test.

Methods	Pedestrian	People	Bicycle	Car	Van	Truck	Tricycle	Awning-Tricycle	Bus	Motor	mAP50 (%)
YOLOv8s	26	13.3	9.3	70.3	36.9	37.2	16.9	18.6	56.3	28.8	31.4
EMFE-YOLO (Ours)	37.6	23.4	13.8	78.4	44.7	39.0	22.0	21.8	58.4	36.8	37.6

**Table 7:** Experimental results of other object detection models on the VisDrone2019-val dataset.

Methods	mAP50 (%)	mAP50:95 (%)	Param (M)
Faster RCNN	37.2	21.9	41.16
CenterNet	33.7	18.8	70.75
YOLOv8n	32.6	18.9	3.2
YOLOv8l	43	26.5	43.7
YOLOv9s	40	24.1	9.6
YOLOv10s	38.5	23.2	8.1
YOLOv11s	39	22.3	9.4
YOLOv12s	39.1	23.6	9.2
TPH-YOLOv5	41.7	24.1	45.4
RT-DETR-R18	42.5	24.5	19.9
YOLO-ERF-L	43.7	23.2	50.2
UAV-YOLOv8	47	29.2	21.5
Drone-YOLO	42.8	25.6	5.35
PVswin-YOLOv8s	43.3	26.4	21.6
TA-YOLO-s	45.4	27.7	13.9
EMFE-YOLO (Ours)	46.9	29.1	3

## Data Availability

The data supporting the results of this study can be obtained from the corresponding author upon reasonable request.
